# Some Diseases of the Male Genital System

**Published:** 1908-02-01

**Authors:** 


### SOME DISEASES OF THE MALE GENITAL SYSTEM.

### VI.—RETAINED TESTIS.

This phrase is used to cover a large number of abnormalities of position of this organ; but these can be divided into two classes—(i) in which the testis has never started its normal passage from the abdomen to the scrotum or has been arrested at some point in it, and (ii) in which the testis has deviated from its normal course. Taking the first variety, four sub-divisions can be recognised according to the point at which the testis has been arrested. Thus the testis may be retained in the abdominal cavity, in which it may be fixed or float freely, this question depending entirely on the length of its peritoneal investment, the mesorchium; or it may descend as far as the internal abdominal ring and remain there without entering the inguinal canal; in this form, which is known as iliac retention, the testis lies upon the iliacus muscle. Or, again, it may be retained actually in the inguinal canal, a position which is not only frequent, but is also important because a testis retained in this position is much exposed to injury, and is thus peculiarly liable to the complications of the condition; lastly, and this is not common, the testis may be retained under the skin at the junction of [illegible]otum and abdominal wall.

The number of hypotheses which have been put forward from time to time to account for retention of the testis in an abnormal position during its transit from the abdomen to the scrotum is literally legion. Is it due to a deficient activity on the part of the normal process which brings about its descent or is it retained by reason of some independent pathological process? The difficulty of answering the question is increased by the fact that our knowledge of the process which brings the testis into the scrotum in the normal course of events is far from being exact. Some would have it that contraction of the gubernaculum testis is the all-important factor; and in support of this view it is argued that contractile muscle fibres can be demonstrated in this structure. Others, however, hold equally strongly that the gubernaculum acts passively by fixing the testis down while the surrounding parts grow away from it; and yet others have suggested that the process is not originally a normal one, but that it should be regarded as a hernia of the testis which has been perpetuated and has become, in the course of time, a constant phenomenon in the human species.

The following are some of the more reasonable hypotheses that have been advanced: (i) That abnormalities in the mesorchium may account for abdominal retention. If this is short or ill-formed the testis is naturally kept in its original position against the posterior abdominal wall, while if it is too long, so that the testis floats free in the abdominal cavity, it is never brought into the neighbourhood of the internal abdominal ring. (ii) That cases of iliac or inguinal retention may be accounted for by contraction or ill-development of some part of the inguinal canal, the internal abdominal ring in the former case and the external in the latter. (iii) That deficiency of the gubernaculum testis should be regarded as the commonest, if not the only, cause of the condition.

Now it has been said that the phrase "retained testis" covers also these conditions in which the testis has been deflected from its normal course, so as to occupy a position which is quite outside its normal path. These positions are three in number—(*a*) Pubic, in which it lies at the root of the penis; (*b*) perineal; and (*c*) crural, in which it leaves the abdomen by the crural ring instead of by the inguinal canal. Let us consider for a moment the anatomy of the inferior attachment of the gubernaculum testis. It divides into three definite fasciculi at its lower end, which are attached respectively to Poupart's ligament, the pubic arch and the fundus scroti. It is supposed by some that these three attachments direct the organ through the inguinal canal, the ligamentous attachment directing it to the internal abdominal ring, the pubic one drawing it along the canal and the scrotal fibres determining its final resting place in the scrotum. It is tempting to suggest that the three varieties of misplaced testis mentioned above are caused by overaction of one or other fasciculus of the gubernaculum. If this view be accepted, it must be supposed that the crural form is the result of excessive contraction of the fibres attached to Poupart's ligament; the pubic, of the band that is inserted into the pubis; and the perineal, of the scrotal part of the structure. At any rate it is easier to attribute the varieties of misplaced testis to abnormalities of the gubernaculum than it is to account for retained testis on the same hypothesis, unless it be supposed that contraction of the gubernaculum is the sole factor in bringing about the normal descent, and that when the testis is retained this structure has, for some reason unexplained, entirely or partially failed to perform its proper function.

A testis retained in an abnormal position renders its possessor liable to a host of complications. For instance, if it be retained in the abdomen a congenital inguinal hernia is almost invariably present. This is easily understood, because a pouch of peritoneum, the processus vaginalis, has been prepared for the testis, and invites the adjacent coils of intestines to descend into it. Again, such a testis may undergo acute torsion of its mesorchium and give rise to symptoms similar to those either of twisted ovarian cyst or of strangulated hernia. It may also become inflamed, secondarily, for instance, to an attack of gonorrhœa, and a few cases have been recorded in which this has set up a fatal peritonitis. If it be retained in the inguinal canal it is exposed to injury because it is fixed in a superficial situation. It is thus liable to constant attacks of inflammation of a slight degree, and such a testis is nearly always found on microscopical examination to present fibrosis of its interstitial tissue and fatty degeneration of its epithelial elements which render it quite functionless.

(*To be continued.*)

